# Supplementary material for: Challenges to pre-migration interventions to prevent human trafficking: Results from a before-and-after learning assessment of training for prospective female migrants in Odisha, India
Source: PLoS One. 2020 Sep 17;15(9):e0238778. doi: 10.1371/journal.pone.0238778 (PMC7498043; doi:10.1371/journal.pone.0238778)
Supplement: S1 File — (PDF) [file pone.0238778.s001.pdf]

PRE-ORIENTATION QUESTIONNAIRE

Block:	Village:

Individual Serial Code:

SWiFT-E Primary Census HH id:	SWiFT-E Primary Survey individual id:

Enumerator ID:	Data entry ID:	Data-entry supervisor ID:

Non-response individual?	Visit number	Date			Time	Reason
		DD	MM	YYYY		
	1					
	2					
	3					

Date of interview:	Interview start time:	Interview end time:

Name of respondent:	
Name of Head of Household in respondent's home:	
Address of respondent:	
Notes on location of respondent's home:<br>[To aid locating respondent for post-orientation survey]	
Alternative place where respondent may be found on date of post-survey<br><br>[To aid locating respondent for post-orientation survey]	
Contact telephone number for respondent:	
Alternative telephone number:<br>(E.g. Family member or neighbour / friend)	

Section 1: Prior exposure to programme messages

1.1			1.2			
**Have you ever received information or advice about worker's rights?** (98 = don't know / don't remember)	**Yes**	**No**	**IF YES: Who gave you this information or advice?** (See codes below. List up to 3)			
				**Other:**		

1.3			1.4			
**Have you ever received information or advice about working away from home?** (98 = don't know / don't remember)	**Yes**	**No**	**IF YES: Who gave you this information or advice?** (See codes below. List up to 3)			
				**Other:**		

CODES: 1 = Spouse, 2 = Natal family 3 = In-law, 4 = Friend, 5 = Neighbour / former neighbour, 6 = AAINA/SEWA, 7 = Other Trade Union, 8 = Other NGO /Gov't scheme worker, 9 = Gov't official, 10 = Religious figure, 11 = Labour contractor/ agent, 12 = Employer, 13 = Other, (specify)

Section 2: Awareness / perceptions of migration opportunities and risks

**2.1**	**Can you tell me some of the main places in India that women from Ganjam go to take up work?**	
	DO NOT SHOW OR READ LIST TO RESPONDENT	*√ all destinations respondent mentions*
i.	Bhubaneshwar / Other destinations within Odisha	
ii.	Mumbai (Bombay) / Maharashtra	
iii.	Kerala	
iv.	Tamil Nadu	
v.	Goa	
vi.	Surat / Gujarat	
vii.	Others:	

**2.2**	**SHOW MAP: Can you show me on this map some of the places women from Ganjam go to take up work?**	Number of places identified on map: (If none then write 0 in box)	

**2.3**			**2.4**		
	**What would you say are the main *benefits* of moving away from home to take up work somewhere else?** (98=don't know) ***DO NOT SHOW OR READ LIST TO RESPONDENT***	**√ all that apply**	**Would you say this benefit is…** (√)		
			Mainly for women	Mainly for men	For men & women
i.	Increased earnings				
ii.	Better quality work				
iii.	Greater employment security				
iv.	Greater independence / autonomy at destination				
v.	Better standard of living / quality of life at destination				
vi.	Escape tensions with spouse				
vii.	Escape tensions with other family members				
viii.	Escape community tensions				
ix.	Escape civic or political unrest / violence				
x.	Other, specify:				

**2.5**			**2.6**		
	**What would you say are the main *dangers/risks* in moving away from home to take up work somewhere else?** ***DO NOT SHOW OR READ LIST TO RESPONDENT***	**√ all that apply**	**Would you say this benefit is…** (√)		
			Mainly for women	Mainly for men	For men & women
i.	Earn too little at destination to cover costs of move				
ii.	Earn too little at destination to fulfil goals (saving, investment, debt-repayment, etc)				

iii.	Being cheated by agent				
iv.	Being cheated by employer				
v.	Sexual assault				
vi.	Physical abuse or assault (not sexual)				
vii.	Forced / Bonded labour situations				
viii.	Injury				
ix.	Illness				
x.	Isolation / loneliness				
xi.	Deterioration of relationship with spouse				
xii.	Deterioration of relationship with children				
xiii.	Social ostracism / stigma on return home				
xiv.	Other, specify:				

2.7	**In your opinion, how old should a girl / woman be before she moves away to take up work elsewhere?** (98=don't know, 88=prefer not to say)	**Age in years**

2.8	**In your opinion, how old should a boy / man be before he moves away to take up work elsewhere?** (98=don't know, 88=prefer not to say)	**Age in years**

Section 3. Awareness / perceptions of safe and risky migration practices

Laxmi was walking home when her neighbour, Dukhi, called to her. Dukhi's brother was visiting. He had news about a good job in Surat. Dukhi and her brother asked Laxmi if she would like to take up the job...

3.1	**What information does Laxmi need to find out before agreeing to take up the job in Surat?** (98 = don't know, 88 = prefer not to say)	
DO NOT SHOW OR READ LIST TO RESPONDENT		***√ all respondent mentions***
i.	The general type of work she will be doing	
ii.	Her specific tasks and responsibilities	
iii.	The name and contact details of any middle men / contractors / placement agents	
iv.	The name and address / location of her employer and workplace	
v.	Whether accommodation is provided by the employer	
vi.	Whether meals are provided by the employer	
vii.	How much her wages / salary will be per day / week / month	
viii.	When she will receive her salary (e.g. daily / weekly / monthly)	
vi.	How she will receive her salary (directly from the employer? From a middleman?)	
x.	What costs she will incur (accommodation / food / uniform, etc.)	
xii.	How many hours per day and days per week she will be expected to work	
xiii.	How many hours of rest she will have per day	
xiv.	The number of weekly paid offs she will have	
xv.	Cultural differences at destination (language spoken / what is the food like)	
xvi.	Other:	

Dukhi's brother offers to give Laxmi an advance on her wages…

3.2	**What do you think? Should Laxmi accept the advance on her wages?** (98 = don't know, 88 = prefer not to say)	Yes (√)	No (√)

3.3	**What are the *advantages* of accepting the advance?** ***DO NOT SHOW OR READ LIST TO RESPONDENT***	**√ all that respondent states**
i.	To cover travel costs	
ii.	To cover setting-up costs at destination	
iii.	To meet food and daily costs in village prior to leaving	
iv.	Strengthen relationship with agent	
v.	To avoid taking loan on worse terms than advance	
vi.	Other, specify:	

3.4	**What are the *dangers or risks* of accepting the advance?** ***DO NOT SHOW OR READ LIST TO RESPONDENT***	**√ all that respondent states**
i.	Increased dependency on the agent	
ii.	Increased dependency on the employer	
iii.	Increased chance of being cheated by the agent	
iv.	Increased chance of being cheated by the employer	
v.	Less freedom to leave job and return home if dissatisfied	
vi.	Other, specify:	

Laxmi bought a mobile phone to take with her when she left Ganjam to work in Surat…

3.5	**Can you think of any reasons to have a mobile phone when working and living away from home?**	
DO NOT SHOW OR READ LIST TO RESPONDENT (98 = don't know, 88 = prefer not to say)		*√ all reasons respondent mentions*
i.	Make and receive calls to keep in touch with family and friends back home	
ii.	Send and receive photos / videos / messages to keep in touch	
iii.	Take photo of agent and / or agent's id	
iv.	Send photo of agent and / or agent's id to family member or other trusted person	
v.	Take photos of important papers to keep a record / use as evidence in a dispute	
vi.	Send photo of important papers to family member or other trusted person	
vii.	Call a relative or friend for help if needed	
viii.	Call an agency or organisation for help if needed (police, NGO or Gov't helpline, etc.)	
ix.	Other:	

3.6	**How strongly do you agree or disagree with the following statements? [READ LIST OUT LOUD]** (98 = don't know, 88 = prefer not to say)	(√ one option only)				
		Strongly agree	Agree	Neither agree nor disagree	Disagree	Strongly disagree
i.	If someone known to me offers to help me move away to find work, I can be sure they will *not* abuse or exploit me					
ii.	There is nothing a woman can do to avoid being cheated, exploited, or abused if she migrates for work					
iii.	Before departing, it is a good idea to check if there is anyone from this village or GP at the destination, and to take their contact details					
iii.	Migrant workers should inform a local official before they move away, in case they have any problems at destination (e.g. their home GP office / Labour office / SHG / Village Head)					
iv.	It is against the law to move to another State in India and take up work					

An employment agent came to Sula's village to recruit people to work in Mumbai. Sula was interested in going but she had heard that agents sometimes deceive and cheat people…

3.7	**What can Sula do to reduce the chance of the agent cheating or deceiving her?**	
DO NOT SHOW OR READ LIST TO RESPONDENT (98 = don't know, 88 = prefer not to say)		*√ all reasons respondent mentions*
i.	Find out the agent's full details (name, address, registration number)	
ii.	Pass the agent's details to a trusted friend or family member	
iii.	Take a photo of the agent and / or the agent's id	
iv.	Send a photo of the agent and / or agent's id to family member or other trusted person	
v.	Refuse to accept an advance payment from the agent	
vi.	Have employer pay wages directly, not via agent	
vii.	Send remittances by bank transfer, not via agent	
ix.	Other:	

Section 4. Attitudes / perceptions to value of women's work and paid domestic work

Two years back, Sita and her husband Babana moved to Mumbai to take up work in construction. Sita and Babana both worked for 8 hours a day, carrying loads and digging. Babana received 200 rupees per day and Sita received 80 rupees per day. Sita didn't question her lower pay…

4.1	**How strongly do you agree or disagree with the following statements? [READ LIST OUT LOUD]** (98 = don't know, 88 = prefer not to say)	(√ one option only)				
		Strongly agree	Agree	Neither agree nor disagree	Disagree	Strongly disagree
i.	"Woman's work" is not as important as "men's work"					
ii.	Men and women should be paid the same for equivalent work					
iii.	Women should not take up employment outside the house					

Soon after Sita and Babana arrived in Mumbai, Sita was offered work as a domestic worker in a family home. When Sita and Babana went back to their village they told people that they were both working as construction workers. Sita didn't want anyone to know that she was working as a domestic worker.

4.2	**How strongly do you agree or disagree with the following statements? [READ LIST OUT LOUD]** (98 = don't know, 88 = prefer not to say)	(√ one option only)				
		Strongly agree	Agree	Neither agree nor disagree	Disagree	Strongly disagree
i.	Sita should feel ashamed to do paid domestic work in someone else's home					
ii.	Paid domestic work is work like any other					
iii.	Paid domestic workers are servants (Chakrani) **not** workers					
iv.	The work paid domestic workers do is essential					
v.	Paid domestic workers should have respect					
vi.	Paid domestic workers have the same rights as all workers					

Section 5. Awareness of workers rights and entitlements

Madhuri was recruited to work as a domestic worker in Kerala. She was not told any details about the job and didn't know what to expect. She left her first employer because she was treated badly. She has found a new employer and wants to know what her rights are before agreeing to begin work.

**5.1**	**What kinds of rights does Madhuri have when she is employed as a domestic worker?**	
DO NOT SHOW OR READ LIST TO RESPONDENT		*√ all areas respondent mentions*
i.	Set pay (set amount of wages / salary)	
ii.	Hours of work (no more than eight hours of work in a single day)	
iii.	Set tasks and responsibilities	
iv.	Regular payment of wages / salary	
v.	Rest periods during the working day	
vi.	Weekly off (paid)	
vii.	Enough & appropriate food, when employer/middleman provides (i.e. "live-in" workers)	
viii.	Appropriate accommodation, when employer/middleman provides (i.e. "live-in" workers)	
ix.	Safety and security at work (and home, when accommodation is provided)	
x.	Medical care arranged and paid for by employer if injured or ill at work	
xi.	Prior notice of dismissal	
xii.	Other:	

**5.2**	**What kinds of responsibilities does Madhuri have when she is employed as a domestic worker?**	
DO NOT SHOW OR READ LIST TO RESPONDENT		*√ all areas respondent mentions*
i.	Beginning work at the agreed time each day	
ii.	Completing agreed tasks diligently	
iii.	Maintaining hygiene at work	
iv.	Respecting the employer's privacy	
v.	Notifying employer if unable to work (due to sickness / family emergency, holiday, etc.)	
vi.	Giving prior notice of resignation	
vii.	Other:	

Madhuri's employer offered to open a savings account for her and to keep her ATM card and bank documents safe. Madhuri thought it was better to open her own savings account so she would have control over her earnings, but was not sure how to do it…

**5.2**	**Where can Madhuri open a savings account?**	
DO NOT SHOW OR READ LIST TO RESPONDENT		√
i.	Bank	
ii.	Post Office	
v.	Other:	

**5.3**	**How strongly do you agree or disagree with the following statements? [READ LIST OUT LOUD]** (98 = don't know, 88 = prefer not to say)	(√ one option only)				
		Strongly agree	Agree	Neither agree nor disagree	Disagree	Strongly disagree
i.	If Madhuri opens a savings account she *must* share her pass book and ATM code with her employer					

ii.	Madhuri cannot open a savings account without her employer's permission					
iii.	Madhuri needs a large sum of money to open a savings account with a bank or post office					
iv.	Madhuri must pay a fee to open a savings account with a bank or post office					
v.	Madhuri can open a bank or post office account *before* leaving to work away from home and access her earnings anywhere in India					
vi.	It is safer and less costly to send earnings home by bank transfer than sending with a person or agent					
vii.	If Madhuri accepts advances on her wages from a middleman or employer she risks being trapped or cheated					

Section 6. Awareness / perceptions of unionisation and organisation

Uma has been working as a domestic worker in Kerala for six years. She visits Ganjam twice a year. Last time she visited home, she was told about a domestic workers' association in Kerala she could join…

**6.1**	**Are you familiar with the idea of a domestic workers association?** (98 = don't know, 88 = prefer not to say)	Yes (√)	No (√)

**6.2**	**Can you think of any reasons why Uma *should* join the domestic workers association?**	
DO NOT SHOW OR READ LIST TO RESPONDENT		*√ all respondent mentions*
i.	Opportunities to socialise with other women / women workers	
ii.	Can find out reliable information about worker's rights	
iii.	Can ask for help from members / organisers to negotiate with employer about pay and working arrangements (hours, tasks, offs, etc.)	
iv.	Can ask for help from members / organisers if treated badly or abused	
v.	Can take part in organised activities to improve paid domestic workers pay and conditions	
vi.	Can take part in organised activities to improve paid domestic social status	
vii.	Other:	

**6.3**	**Can you think of any reasons why Uma should *not* join the domestic workers association?**	
DO NOT SHOW OR READ LIST TO RESPONDENT		*√ all respondent mentions*
i.	No personal benefit from joining	
ii.	Will take up too much time	
iii.	Too expensive to join	
iv.	Other members may cheat / take advantage	
v.	Other people may think badly of her if she joins	
vi.	Other:	

**6.4**	**Overall, what do you think? Should Uma join the domestic workers association?** (98 = don't know)	Yes (√)	No (√)

Section 7. Intentions to migrate

**7.1**	**As of now, do you have any thoughts about moving away from this village to work somewhere else for a time?** (98 = don't know, 88 = prefer not to say)	Yes (√)	No (√)

IF 7.1 = NO				
**7.2**	**Are there any reasons why you would <u>not</u> consider moving away from this village to work somewhere else for a time?** (See code list. List up to 3)			
Other:				

IF 7.1 = YES				
**7.3**	**Why are you considering moving away from this village to work somewhere else for a time?** (See code list. List up to 3)			
Other:				

IF 7.1 = YES				
**7.4.**				**7.5**
**Have you thought about… [READ LIST OUT LOUD]** (98 = don't know, 88 = prefer not to say)		Yes	No	**If 7.4 = YES: Can you tell me…**
i.	Where you would move away to			
ii.	How long you would move away for			
iii.	How to reach your destination			
iv.	How to find work at your destination			
v.	What type of work you would most want to do			
vi.	What type of work you would **not** want to do			
vii.	How you can stay healthy			
viii.	What you would do if you were ill or injured			
ix.	How much money you would need to migrate and live away from home			
x.	How much money you could earn in total			
xi.	Where you will keep your earnings when you receive them			
xii.	How your earnings will be spent			
xiii.	How much of your earnings you will send to your family back home			
xiv.	How much money you could save in total			
xv.	What you would use any savings for			
xvi.	What you would do if you were badly treated or cheated on your way to your destination			
xvii.	What you would do if you were badly treated or cheated at destination			
xviii.	What you would do if you were prevented by your employer or recruiter from leaving your job when you wanted to			

Section 8. Awareness of existing livelihood & welfare schemes

**8.1**	**Have you heard of any of the following types of government / NGO schemes?**<br>**READ LIST TO PARTICIPANTS** (0 = no, 1 = yes)

RSBY / BKKY (or other healthcare)	Indira Awas Yojana (or other housing)	Job / labour card	Vocational training programmes	NREGA	Pension (old age, widow, disability, etc.)	Land patta	Forest patta	Swachh Bharat
**Any others?**								

8.2	**Have you enrolled in any of the following government / NGO schemes?** **READ LIST TO PARTICIPANTS** (0 = no, 1 = yes)

RSBY / BKKY (or other healthcare)	Indira Awas Yojana (or other housing)	Job / labour card	Vocational training programmes	NREGA	Pension (old age, widow, disability, etc.)	Land patta	Forest patta	Swachh Bharat
**Any others?**								

8.3	**As of now, do you have any plans to apply for any of the following government / NGO schemes?** **READ LIST TO PARTICIPANTS** (0 = no, 1 = yes)

RSBY / BKKY (or other healthcare)	Indira Awas Yojana (or other housing)	Job / labour card	Vocational training programmes	NREGA	Pension (old age, widow, disability, etc.)	Land patta	Forest patta	Swachh Bharat
**Any others?**								

Section 9. Reasons for attending the WiF two-day pre-decision orientation sessions

**9.1**	**What is your *main* reason for signing up to attend the Work in Freedom two-day orientation?**	
DO NOT SHOW OR READ LIST TO RESPONDENT		*√ one option only*
i.	To find out about opportunities to migrate for work	
ii.	To find out about the benefits of migrating for work	
iii.	To find out about the risks or dangers of migrating for work	
iv.	To find out how to migrate safely and avoid being cheated or badly treated	
v.	To find out what migrant workers should do if they are cheated or badly treated	
vi.	To find out about sources of help and support available to migrant workers	
vii.	To find out about women's rights	
viii.	To find out about women's health	
xi.	To find out about worker's rights	
x.	To find out about banking, savings, and loans	
xi.	To find out about livelihood and welfare schemes available locally	
xii.	To find out about livelihood and welfare schemes available to migrant workers	
xiii.	Other reason:	

**9.2**	**Are you attending the Work in Freedom two-day orientation for yourself or for someone else?**	
DO NOT SHOW OR READ LIST TO RESPONDENT		*√ one option only*
i.	Myself only	
ii.	Myself & my husband	
iii.	Myself & other male family member(s)	
iv.	Myself & other female family member(s)	
v.	My husband only	
vi.	My son(s) only	
vii.	My daughter(s) only	
viii.	Other male family member(s)	
xi.	Other female family member(s)	
viii.	Male non-family member(s)	
xi.	Female non-family member(s)	

Section 10. Respondent characteristics

**10.1** Age in years:							
**10.2** Marital status:		1=Never married, 2=Currently Married, 3=Divorced/Separated, 4=Abandoned					
**10.3** Number of sons		Sons' ages	**10.3a**	**10.3b**	**10.3c**	**10.3d**	**10.3e**
**10.4** Number of daughters		Daughters' ages	**10.4a**	**10.4b**	**10.4c**	**10.4d**	**10.4e**
**10.5** Caste name (write in words):							
**10.6** Caste code:		1=SC, 2=ST, 3=OBC, 4=Others					
**10.7** Religion:		1=Hindu, 2=Muslim, 3=Christian, 4=Buddhist, 5=Other (specify)					
**10.8** Education level:		0=no formal education, 1 – 12=Highest class completed, 13=University or equivalent					
**10.9** Literacy:		0=Cannot read or write in any language,<br>1=Can read Odiya,<br>2=Can write in Odiya,<br>3=Can read and write in Odiya,<br>4=Can read Odiya and another language,<br>5=Can write in Odiya and another language,<br>6=Can read and write in Odiya and another language					
**10.10** Number of languages spoken:							
**10.11** Main occupation:		(See occupation codes, below)					
**10.12** Secondary occupation:		(See occupation codes, below)					
**10.13** Prior experience of migration?		1=yes, 2=no, 98 = don't know, 88 = prefer not to say					
Occupations at destination:	**10.14**	**10.14a**	**10.14b**	(See occupation codes, below. List up to 3)			

**10.15** Husband's age in years:				
**10.16** Husband's education level:		0=no formal education, 1–12=Highest class completed, 13=University/equivalent		
**10.17** Husband's main occupation:		(See occupation codes, below)		
**10.18** Husband's secondary occupation:				
**10.19** Husband has prior experience of migration?		1=yes, 2=no, 98 = don't know, 88 = prefer not to say		
Husband's occupations at destination:	**10.20**	**10.20a**	**10.20b**	(See occupation codes, below. List up to 3)

**10.21** Main source of household income or subsistenc		(See income source codes, below)	
**10.22** Yearly household income (approx.) INR			
**10.23** Total value of outstanding household loans (approx.) INR			
**10.24** Household land owned:		**10.25** Total number of bharana owned (0 if none)	
**10.26** Type of HH ration card?		1 = BPL, 2 = APL, 3 = Antyodaya, 4 = other, 98 = don't know, 88 = prefer not to say	
**10.27** Household hires in labour:		1=yes, 2=no, 98 = don't know, 88 = prefer not to say	

	Female		Male	
	Aged under 15	Aged 15 +	Aged under 15	Aged 15 +
**Total** number of HH members	**10.28f1**	**10.28f2**	**10.28m1**	**10.28m2**
Number of HH members who currently have an Aadhaar card registered in their name?	**10.29f1**	**10.29f2**	**10.29m1**	**10.29m2**
Number of HH members who do **any** income-earning labour	**10.30f1**	**10.30f2**	**10.30m1**	**10.30m2**
Number of HH members in full-time education	**10.31f1**	**10.31f2**	**10.31m1**	**10.31m2**
Number of HH members who cannot do income-earning work due to illness / disability / old-age	**10.32f1**	**10.32f2**	**10.32m1**	**10.32m2**

Number of HH members who have **ever** moved away to take up work	**10.33f1**	**10.33f2**	**10.33m1**	**10.33m2**
Number of HH members who are **currently** living or working elsewhere	**10.34f1**	**10.34f2**	**10.34m1**	**10.34m2**
Number of HH members who are **currently** considering moving away to work elsewhere	**10.35f1**	**10.35f2**	**10.35m1**	**10.35m2**

CODE LIST: Pre-Orientation Survey

Question 7.2: Are there any reasons why you would not consider moving away from this village to work somewhere else for a time?

1	Has sufficient income from paid work in the village and surrounding area
2	Has land in the village that provides sufficient livelihood
3	No one to take care of children in case of migration
4	No one to take care of household duties in case of migration
5	Fear of losing property right in the village
6	Own ill-health prevents migration
7	Family member's ill-health prevents migration
8	Spouse will not give permission for migration
9	Other family member will not give permission for migration
10	Cannot afford costs of migrating
11	Does not know how to go about migrating
12	Wary of being cheated or exploited by labour contractors
13	Wary of being cheated or exploited by employer at destination
14	Fear of bonded labour
15	Fear of traffickers
16	Work not available at destination
17	Low wages at destination
18	Poor conditions of work
19	Loneliness/home sickness
20	High cost of living at destination
21	Poor conditions of residence at destination
22	Fear of physical insecurity at destination
23	Fear of conflict at destination
24	Fear of children becoming estranged due to separation
25	Fear of spouse becoming estranged due to long separation
26	Fear of loneliness/home sickness
27	Fear of social ostracism / stigma at destination
28	Fear of social ostracism / stigma on return to village
29	Other (specify)

Question 7.3: Why you are considering moving away from this village to work somewhere else for a time?

1	Chronic/regular inability to meet basic needs in village
2	Temporary/seasonal inability to meet basic needs in village
3	Chronic/regular absence of employment in village and surrounding area
4	Temporary / seasonal absence of employment in village and surrounding area
5	Loss of job / business failure
6	Sudden loss of income
7	To finance agriculture
8	To finance non-farm business
9	To seek better paid employment
10	Attractive offer by labour intermediary/contractor
11	To seek better quality employment
12	To seek employment commensurate to education/skills
13	To enhance standard of living / quality of life
14	Unable to meet loan repayments without migrant income
15	Condition of advance / loan agreement
16	Displacement due to weather / natural event
17	Displacement due to political / civic unrest
18	Displacement from land due to government acquisition
19	Displacement from land due to private acquisition
20	Communal tensions
21	Caste tensions
22	Domestic violence
23	Other tensions with spouse (not domestic violence)
24	Other tensions with natal family members (not domestic violence)
25	Other tensions with in-laws (not domestic violence)
26	Violent conflict with other households / individuals
27	Social ostracism / stigma
28	Appeal of greater independence
29	Appeal of adventure
30	Appeal of new challenges
31	Self advancement
32	Advancement of the household
33	Children's advancement
34	To raise dowry
35	To (help) care for immediate family members at destination
36	To (help) care for other relations at destination
37	To attend educational institution
38	Accompanying spouse
39	Accompanying other family member
40	For marriage
41	Other (specify)

Section 8: Main Occupation, Secondary Occupation, & Occupations at destination (for self and husband)

1	Agricultural labourer	21	Mechanic
2	Construction labourer	22	Paid domestic work (cleaning)
3	Mason	23	Nurse or paramedic or midwife
4	Carpenter	24	Security guard
5	Farmer / cultivator	25	Carer, paid (child and elder care, etc.)
6	Cattle rearing and herding	26	Administrator
7	Fisherman / fisherwoman	27	Religious work (priest, etc)
8	Artisan (pottery, textiles, metal-work, etc.)	28	Sanitation worker
9	Minor forest producer / gatherer	29	Engineer
10	Hawker	30	Manager
11	Trader / merchant	31	Doctor
12	Quarrying / other mining work	32	Lawyer
13	Tailor	33	Moneylender
14	Driver / taxi-driver / courier	34	Tours and tourism employees
15	Teacher	35	*Undertakes household chores*
16	Tutor	36	*Undertakes HH chores & income-substituting activities*
17	Cook or chef	37	*Oversees household chores*
18	Janitor	38	In full-time education
19	Gardener	39	Beggar
20	Electrician	40	Other, specify

Section 8: Main source of household income or subsistence:

1	Casual / daily wage labour: Agricultural
2	Casual / daily wage labour: Non-agricultural
3	Own Account Business: Agricultural
4	Own Account Business: Non-Agricultural
5	Self-employed with hired workers: Agricultural
6	Self-employed with hired workers: Non-agricultural
7	Regular salaried employment

LONDON SCHOOL of HYGIENE &TROPICAL MEDICINE

POST-ORIENTATION QUESTIONNAIRE

Block:	Village:

Individual Serial Code:

SWiFT-E Primary Census HH id:	SWiFT-E Primary Survey individual id:

Enumerator ID:	Data entry ID:	Data-entry supervisor ID:

Non-response individual?	Visit number	Date			Time	Reason
		DD	MM	YYYY		
	1					
	2					
	3					

Date of interview:	Interview start time:	Interview end time:

Name of respondent:	
Name of Head of Household in respondent's home:	
Address of respondent:	

Section 1: Attendance at WiF pre-departure orientation sessions

**1.1**	**Did you attend the AAINA training sessions about women and migration?** (88 = prefer not to say)	Yes (√)	No (√)

**IF 1.1 = YES**		
**1.2**	**Did you attend all of the sessions or only some?** ***DO NOT SHOW OR READ LIST TO RESPONDENT***	*√ one option only*
i.	Attended part of day one: *Under 3 hours*	
ii.	Attended part of day one: *Over 3 hours*	
iii.	Attended all of day one	
iv.	Attended all of day one and part of day two	
v.	Attended all of day one and all of day two	

**IF 1.1 = NO OR 1.2 IS PARTIAL**		
**1.3**	**Was there some reason why you could not attend [all of] the sessions?** ***DO NOT SHOW OR READ LIST TO RESPONDENT*** (88 = prefer not to say)	*√ all reasons respondent gives*
i.	Could not take time away from paid / income-earning work	
ii.	Could not take time away from unpaid household work / childcare / elder care	
iii.	Was ill and could not attend	
iv.	Child or other family member was ill so could not attend	
v.	Prohibited by spouse	
vi.	Prohibited by other family member	

vii.	Was not interested / did not think it was relevant to me	
	Other:	

Section 2: Awareness / perceptions of migration opportunities and risks

2.1	**Can you tell me some of the main places in India that women from Ganjam go to take up work?**	
	DO NOT SHOW OR READ LIST TO RESPONDENT	*√ all destinations respondent mentions*
i.	Bhubaneshwar / Other destinations within Odisha	
ii.	Mumbai (Bombay) / Maharashtra	
iii.	Kerala	
iv.	Tamil Nadu	
v.	Goa	
vi.	Surat / Gujarat	
vii.	Others:	

2.2	**SHOW MAP: Can you show me on this map some of the places women from Ganjam go to take up work?**	Number of places identified on map: (If none then write 0 in box)	

2.3			2.4		
**What would you say are the main *benefits* of moving away from home to take up work somewhere else?** (98=don't know) ***DO NOT SHOW OR READ LIST TO RESPONDENT***		**√ all that apply**	**Would you say this benefit is…** (√)		
			Mainly for women	Mainly for men	For men & women
i.	Increased earnings				
ii.	Better quality work				
iii.	Greater employment security				
iv.	Greater independence / autonomy at destination				
v.	Better standard of living / quality of life at destination				
vi.	Escape tensions with spouse				
vii.	Escape tensions with other family members				
viii.	Escape community tensions				
ix.	Escape civic or political unrest / violence				
x.	Other, specify:				

2.5			2.6		
**What would you say are the main *dangers/risks* in moving away from home to take up work somewhere else?** ***DO NOT SHOW OR READ LIST TO RESPONDENT***		**√ all that apply**	**Would you say this benefit is…** (√)		
			Mainly for women	Mainly for men	For men & women
i.	Earn too little at destination to cover costs of move				
ii.	Earn too little at destination to fulfil goals (saving, investment, debt-repayment, etc)				
iii.	Being cheated by agent				
iv.	Being cheated by employer				
v.	Sexual assault				
vi.	Physical abuse or assault (not sexual)				
vii.	Forced / Bonded labour situations				
viii.	Injury				
ix.	Illness				
x.	Isolation / loneliness				
xi.	Deterioration of relationship with spouse				
xii.	Deterioration of relationship with children				

xiii.	Social ostracism / stigma on return home				
xiv.	Other, specify:				

2.7	**In your opinion, how old should a girl / woman be before she moves away to take up work elsewhere?** (98=don't know, 88=prefer not to say)	**Age in years**

2.8	**In your opinion, how old should a boy / man be before he moves away to take up work elsewhere?** (98=don't know, 88=prefer not to say)	**Age in years**

Section 3. Awareness / perceptions of safe and risky migration practices

Laxmi was walking home when her neighbour, Dukhi, called to her. Dukhi's brother was visiting. He had news about a good job in Surat. Dukhi and her brother asked Laxmi if she would like to take up the job...

3.1	**What information does Laxmi need to find out before agreeing to take up the job in Surat?** (98 = don't know, 88 = prefer not to say)	
	DO NOT SHOW OR READ LIST TO RESPONDENT	***√ all respondent mentions***
i.	The general type of work she will be doing	
ii.	Her specific tasks and responsibilities	
iii.	The name and contact details of any middle men / contractors / placement agents	
iv.	The name and address / location of her employer and workplace	
v.	Whether accommodation is provided by the employer	
vi.	Whether meals are provided by the employer	
vii.	How much her wages / salary will be per day / week / month	
viii.	When she will receive her salary (e.g. daily / weekly / monthly)	
vi.	How she will receive her salary (directly from the employer? From a middleman?)	
x.	What costs she will incur (accommodation / food / uniform, etc.)	
xii.	How many hours per day and days per week she will be expected to work	
xiii.	How many hours of rest she will have per day	
xiv.	The number of weekly paid offs she will have	
xv.	Cultural differences at destination (language spoken / what is the food like)	
xvi.	Other:	

Dukhi's brother offers to give Laxmi an advance on her wages…

3.2	**What do you think? Should Laxmi accept the advance on her wages?** (98 = don't know, 88 = prefer not to say)	Yes (√)	No (√)

3.3	**What are the *advantages* of accepting the advance?** ***DO NOT SHOW OR READ LIST TO RESPONDENT***	**√ all that respondent states**
i.	To cover travel costs	
ii.	To cover setting-up costs at destination	
iii.	To meet food and daily costs in village prior to leaving	
iv.	Strengthen relationship with agent	
v.	To avoid taking loan on worse terms than advance	
vi.	Other, specify:	

3.4	**What are the *dangers or risks* of accepting the advance?** ***DO NOT SHOW OR READ LIST TO RESPONDENT***	**√ all that respondent states**
i.	Increased dependency on the agent	

ii.	Increased dependency on the employer	
iii.	Increased chance of being cheated by the agent	
iv.	Increased chance of being cheated by the employer	
v.	Less freedom to leave job and return home if dissatisfied	
vi.	Other, specify:	

Laxmi bought a mobile phone to take with her when she left Ganjam to work in Surat…

3.5	Can you think of any reasons to have a mobile phone when working and living away from home?	
	DO NOT SHOW OR READ LIST TO RESPONDENT<br>(98 = don't know, 88 = prefer not to say)	*√ all reasons respondent mentions*
i.	Make and receive calls to keep in touch with family and friends back home	
ii.	Send and receive photos / videos / messages to keep in touch	
iii.	Take photo of agent and / or agent's id	
iv.	Send photo of agent and / or agent's id to family member or other trusted person	
v.	Take photos of important papers to keep a record / use as evidence in a dispute	
vi.	Send photo of important papers to family member or other trusted person	
vii.	Call a relative or friend for help if needed	
viii.	Call an agency or organisation for help if needed (police, NGO or Gov't helpline, etc.)	
ix.	Other:	

3.6	**How strongly do you agree or disagree with the following statements? [READ LIST OUT LOUD]**<br>(98 = don't know, 88 = prefer not to say)	(√ one option only)				
		Strongly agree	Agree	Neither agree nor disagree	Disagree	Strongly disagree
i.	If someone known to me offers to help me move away to find work, I can be sure they will *not* abuse or exploit me					
ii.	There is nothing a woman can do to avoid being cheated, exploited, or abused if she migrates for work					
iii.	Before departing, it is a good idea to check if there is anyone from this village or GP at the destination, and to take their contact details					
iii.	Migrant workers should inform a local official before they move away, in case they have any problems at destination (e.g. their home GP office / Labour office / SHG / Village Head)					
iv.	It is against the law to move to another State in India and take up work					

An employment agent came to Sula's village to recruit people to work in Mumbai. Sula was interested in going but she had heard that agents sometimes deceive and cheat people…

3.7	What can Sula do to reduce the chance of the agent cheating or deceiving her?	
	DO NOT SHOW OR READ LIST TO RESPONDENT<br>(98 = don't know, 88 = prefer not to say)	*√ all reasons respondent mentions*
i.	Find out the agent's full details (name, address, registration number)	
ii.	Pass the agent's details to a trusted friend or family member	
iii.	Take a photo of the agent and / or the agent's id	
iv.	Send a photo of the agent and / or agent's id to family member or other trusted person	

v.	Refuse to accept an advance payment from the agent	
vi.	Have employer pay wages directly, not via agent	
vii.	Send remittances by bank transfer, not via agent	
ix.	Other:	

Section 4. Attitudes / perceptions to value of women's work and paid domestic work

Two years back, Sita and her husband Babana moved to Mumbai to take up work in construction. Sita and Babana both worked for 8 hours a day, carrying loads and digging. Babana received 200 rupees per day and Sita received 80 rupees per day. Sita didn't question her lower pay…

4.1	**How strongly do you agree or disagree with the following statements? [READ LIST OUT LOUD]** (98 = don't know, 88 = prefer not to say)	(√ one option only)				
		Strongly agree	Agree	Neither agree nor disagree	Disagree	Strongly disagree
i.	"Woman's work" is not as important as "men's work"					
ii.	Men and women should be paid the same for equivalent work					
iii.	Women should not take up employment outside the house					

Soon after Sita and Babana arrived in Mumbai, Sita was offered work as a domestic worker in a family home. When Sita and Babana went back to their village they told people that they were both working as construction workers. Sita didn't want anyone to know that she was working as a domestic worker.

4.2	**How strongly do you agree or disagree with the following statements? [READ LIST OUT LOUD]** (98 = don't know, 88 = prefer not to say)	(√ one option only)				
		Strongly agree	Agree	Neither agree nor disagree	Disagree	Strongly disagree
i.	Sita should feel ashamed to do paid domestic work in someone else's home					
ii.	Paid domestic work is work like any other					
iii.	Paid domestic workers are servants (Chakrani) **not** workers					
iv.	The work paid domestic workers do is essential					
v.	Paid domestic workers should have respect					
vi.	Paid domestic workers have the same rights as all workers					

Section 5. Awareness of workers rights and entitlements

Madhuri was recruited to work as a domestic worker in Kerala. She was not told any details about the job and didn't know what to expect. She left her first employer because she was treated badly. She has found a new employer and wants to know what her rights are before agreeing to begin work.

5.1	**What kinds of rights does Madhuri have when she is employed as a domestic worker?**	
DO NOT SHOW OR READ LIST TO RESPONDENT		*√ all areas respondent mentions*
i.	Set pay (set amount of wages / salary)	

ii.	Hours of work (no more than eight hours of work in a single day)	
iii.	Set tasks and responsibilities	
iv.	Regular payment of wages / salary	
v.	Rest periods during the working day	
vi.	Weekly off (paid)	
vii.	Enough & appropriate food, when employer/middleman provides (i.e. "live-in" workers)	
viii.	Appropriate accommodation, when employer/middleman provides (i.e. "live-in" workers)	
ix.	Safety and security at work (and home, when accommodation is provided)	
x.	Medical care arranged and paid for by employer if injured or ill at work	
xi.	Prior notice of dismissal	
xii.	Other:	

**5.2**	**What kinds of responsibilities does Madhuri have when she is employed as a domestic worker?**	
DO NOT SHOW OR READ LIST TO RESPONDENT		*√ all areas respondent mentions*
i.	Beginning work at the agreed time each day	
ii.	Completing agreed tasks diligently	
iii.	Maintaining hygiene at work	
iv.	Respecting the employer's privacy	
v.	Notifying employer if unable to work (due to sickness / family emergency, holiday, etc.)	
vi.	Giving prior notice of resignation	
vii.	Other:	

Madhuri's employer offered to open a savings account for her and to keep her ATM card and bank documents safe. Madhuri thought it was better to open her own savings account so she would have control over her earnings, but was not sure how to do it…

**5.2**	**Where can Madhuri open a savings account?**	
DO NOT SHOW OR READ LIST TO RESPONDENT		*√*
i.	Bank	
ii.	Post Office	
v.	Other:	

**5.3**	**How strongly do you agree or disagree with the following statements? [READ LIST OUT LOUD]** (98 = don't know, 88 = prefer not to say)	(√ one option only)				
		Strongly agree	Agree	Neither agree nor disagree	Disagree	Strongly disagree
i.	If Madhuri opens a savings account she *must* share her pass book and ATM code with her employer					
ii.	Madhuri cannot open a savings account without her employer's permission					
iii.	Madhuri needs a large sum of money to open a savings account with a bank or post office					
iv.	Madhuri must pay a fee to open a savings account with a bank or post office					
v.	Madhuri can open a bank or post office account *before* leaving to work away from home and access her earnings anywhere in India					
vi.	It is safer and less costly to send earnings home by bank transfer than sending with a person or agent					

vii.	If Madhuri accepts advances on her wages from a middleman or employer she risks being trapped or cheated					

Section 6. Awareness / perceptions of unionisation and organisation

Uma has been working as a domestic worker in Kerala for six years. She visits Ganjam twice a year. Last time she visited home, she was told about a domestic workers' association in Kerala she could join…

**6.1**	**Are you familiar with the idea of a domestic workers association?** (98 = don't know, 88 = prefer not to say)	Yes (√)	No (√)

**6.2**	**Can you think of any reasons why Uma *should* join the domestic workers association?**	
DO NOT SHOW OR READ LIST TO RESPONDENT		*√ all respondent mentions*
i.	Opportunities to socialise with other women / women workers	
ii.	Can find out reliable information about worker's rights	
iii.	Can ask for help from members / organisers to negotiate with employer about pay and working arrangements (hours, tasks, offs, etc.)	
iv.	Can ask for help from members / organisers if treated badly or abused	
v.	Can take part in organised activities to improve paid domestic workers pay and conditions	
vi.	Can take part in organised activities to improve paid domestic social status	
vii.	Other:	

**6.3**	**Can you think of any reasons why Uma should *not* join the domestic workers association?**	
DO NOT SHOW OR READ LIST TO RESPONDENT		*√ all respondent mentions*
i.	No personal benefit from joining	
ii.	Will take up too much time	
iii.	Too expensive to join	
iv.	Other members may cheat / take advantage	
v.	Other people may think badly of her if she joins	
vi.	Other:	

**6.4**	**Overall, what do you think? Should Uma join the domestic workers association?** (98 = don't know)	Yes (√)	No (√)

Section 7. Intentions to migrate

**7.1**	**As of now, do you have any thoughts about moving away from this village to work somewhere else for a time?** (98 = don't know, 88 = prefer not to say)	Yes (√)	No (√)

**IF 7.1 = NO**				
**7.2**	**Are there any reasons why you would <u>not</u> consider moving away from this village to work somewhere else for a time?** (See code list below. List up to 3)			
Other:				

**IF 7.1 = YES**				
**7.3**	**Why are you considering moving away from this village to work somewhere else for a time?** (See code list below. List up to 3)			
Other:				

**IF 7.1 = YES**				
**7.4.**				**7.5**
**Have you thought about… [READ LIST OUT LOUD]** (98 = don't know, 88 = prefer not to say)		Yes	No	**If 7.4 = YES: Can you tell me…**
i.	Where you would move away to			
ii.	How long you would move away for			
iii.	How to reach your destination			
iv.	How to find work at your destination			
v.	What type of work you would most want to do			
vi.	What type of work you would **not** want to do			
vii.	How you can stay healthy			
viii.	What you would do if you were ill or injured			
ix.	How much money you would need to migrate and live away from home			
x.	How much money you could earn in total			
xi.	Where you will keep your earnings when you receive them			
xii.	How your earnings will be spent			
xiii.	How much of your earnings you will send to your family back home			
xiv.	How much money you could save in total			
xv.	What you would use any savings for			
xvi.	What you would do if you were badly treated or cheated on your way to your destination			
xvii.	What you would do if you were badly treated or cheated at destination			
xviii.	What you would do if you were prevented by your employer or recruiter from leaving your job when you wanted to			

**7.6**	Do you have prior experience of migration?		1=yes, 2=no, 98 = don't know, 88 = prefer not to say		
**7.7**	If 7.6 Yes: Occupations at destination:				(See occupation codes, below. List up to 3)

**7.8**	Female		Male	
	Aged under 15	Aged 15 +	Aged under 15	Aged 15 +
Number of HH members who have **ever** moved away to take up work				
Number of HH members who are **currently** living or working elsewhere				
Number of HH members who are **currently** considering moving away to work elsewhere				

Section 8. Awareness of existing livelihood & welfare schemes

**8.1**	**Have you heard of any of the following types of government / NGO schemes?**<br>**READ LIST TO PARTICIPANTS** (0 = no, 1 = yes)

RSBY / BKKY (or other healthcare)	Indira Awas Yojana (or other housing)	Job / labour card	Vocational training programmes	NREGA	Pension (old age, widow, disability, etc.)	Land patta	Forest patta	Swachh Bharat
**Any others?**								

8.2	**As of now, do you have any plans to apply for any of the following government / NGO schemes? READ LIST TO PARTICIPANTS** (0 = no, 1 = yes)							
RSBY / BKKY (or other healthcare)	Indira Awas Yojana (or other housing)	Job / labour card	Vocational training programmes	NREGA	Pension (old age, widow, disability, etc.)	Land patta	Forest patta	Swachh Bharat
**Any others?**								

Section 9. Impressions of orientation delivery and content

9.1	**How strongly do you agree or disagree with the following statements about the AAINA orientation on women and migration? [READ LIST OUT LOUD]** (98 = don't know, 88 = prefer not to say)	(√ one option only)				
		Strongly agree	Agree	Neither agree nor disagree	Disagree	Strongly disagree
i.	I could follow and understand the information I received					
ii.	I learnt information about the risks and dangers of migration that I didn't know before					
iii.	I learnt information about how to migrate safely that I didn't know before					
iv.	I learnt information about workers' rights that I didn't know before					
v.	I learnt information about women's rights that I didn't know before					
vi.	I learnt information about women's anatomy that I didn't know before					
vii.	I learnt information about good health that I didn't know before					
viii.	I learnt information about finance and banking that I didn't know before					
ix ~~viii.~~	I learnt information about government livelihood / welfare schemes that I didn't know before					
x ~~ix.~~	I learnt information about how to use a mobile phone that I didn't know before					
xi ~~x.~~	The information I received is relevant to my situation					
xii ~~xi.~~	The information I received is relevant to the situation of my family members					
xiii ~~xii.~~	I enjoyed taking part in the learning activities					
xiv ~~xiii.~~	If a woman asked me if she should attend the orientation, I would recommend that she do					

9.2	**What did you like *most* about the AAINA orientation on women and migration?**

9.3	**What did you like *least* about the AAINA orientation on women and migration?**

9.4	**Do you have any ideas about to make the training better in future?**

CODE LIST: Post-Orientation Survey

Question 7.2: Are there any reasons why you would not consider moving away from this village to work somewhere else for a time?

1	Has sufficient income from paid work in the village and surrounding area
2	Has land in the village that provides sufficient livelihood
3	No one to take care of children in case of migration
4	No one to take care of household duties in case of migration
5	Fear of losing property right in the village
6	Own ill-health prevents migration
7	Family member's ill-health prevents migration
8	Spouse will not give permission for migration
9	Other family member will not give permission for migration
10	Cannot afford costs of migrating
11	Does not know how to go about migrating
12	Wary of being cheated or exploited by labour contractors
13	Wary of being cheated or exploited by employer at destination
14	Fear of bonded labour
15	Fear of traffickers
16	Work not available at destination
17	Low wages at destination
18	Poor conditions of work
19	Loneliness/home sickness
20	High cost of living at destination
21	Poor conditions of residence at destination
22	Fear of physical insecurity at destination
23	Fear of conflict at destination
24	Fear of children becoming estranged due to separation
25	Fear of spouse becoming estranged due to long separation
26	Fear of loneliness/home sickness
27	Fear of social ostracism / stigma at destination
28	Fear of social ostracism / stigma on return to village
29	Other (specify)

Question 7.3: Why you are considering moving away from this village to work somewhere else for a time?

1	Chronic/regular inability to meet basic needs in village
2	Temporary/seasonal inability to meet basic needs in village
3	Chronic/regular absence of employment in village and surrounding area
4	Temporary / seasonal absence of employment in village and surrounding area
5	Loss of job / business failure
6	Sudden loss of income
7	To finance agriculture
8	To finance non-farm business
9	To seek better paid employment
10	Attractive offer by labour intermediary/contractor
11	To seek better quality employment
12	To seek employment commensurate to education/skills
13	To enhance standard of living / quality of life
14	Unable to meet loan repayments without migrant income
15	Condition of advance / loan agreement
16	Displacement due to weather / natural event
17	Displacement due to political / civic unrest
18	Displacement from land due to government acquisition
19	Displacement from land due to private acquisition
20	Communal tensions
21	Caste tensions
22	Domestic violence
23	Other tensions with spouse (not domestic violence)
24	Other tensions with natal family members (not domestic violence)
25	Other tensions with in-laws (not domestic violence)
26	Violent conflict with other households / individuals
27	Social ostracism / stigma
28	Appeal of greater independence
29	Appeal of adventure
30	Appeal of new challenges
31	Self advancement
32	Advancement of the household
33	Children's advancement
34	To raise dowry
35	To (help) care for immediate family members at destination
36	To (help) care for other relations at destination
37	To attend educational institution
38	Accompanying spouse
39	Accompanying other family member
40	For marriage
41	Other (specify)

Question 7.6: Occupations at destination

1	Agricultural labourer	21	Mechanic
2	Construction labourer	22	Paid domestic work (cleaning)
3	Mason	23	Nurse or paramedic or midwife
4	Carpenter	24	Security guard
5	Farmer / cultivator	25	Carer, paid (child and elder care, etc.)
6	Cattle rearing and herding	26	Administrator
7	Fisherman / fisherwoman	27	Religious work (priest, etc)
8	Artisan (pottery, textiles, metal-work, etc.)	28	Sanitation worker
9	Minor forest producer / gatherer	29	Engineer
10	Hawker	30	Manager
11	Trader / merchant	31	Doctor
12	Quarrying / other mining work	32	Lawyer
13	Tailor	33	Moneylender
14	Driver / taxi-driver / courier	34	Tours and tourism employees
15	Teacher	35	*Undertakes household chores*
16	Tutor	36	*Undertakes HH chores & income-substituting activities*
17	Cook or chef	37	*Oversees household chores*
18	Janitor	38	In full-time education
19	Gardener	39	Beggar
20	Electrician	40	Other, specify
